# Extended morphological processing: a practical method for automatic spot detection of biological markers from microscopic images

**DOI:** 10.1186/1471-2105-11-373

**Published:** 2010-07-08

**Authors:** Yoshitaka Kimori, Norio Baba, Nobuhiro Morone

**Affiliations:** 1 Japan Association for the Advancement of Medical Equipment, Hongo 3-42-6, Bunkyo-ku, Tokyo, 113-0033, Japan; 2 Department of Ultrastructural Research, National Institute of Neuroscience, National Center of Neurology and Psychiatry, Ogawahigashi-cho 4-1-1, Kodaira, Tokyo, 187-8502, Japan; 3 Center for Novel Science Initiatives, National Institutes of Natural Sciences, Toranomon 4-3-13, Minato-ku, Tokyo, 105-0001, Japan; 4 Faculty of informatics, Kogakuin University, Nishi-shinjuku 1-24-2, Shinjuku-ku, Tokyo, 163-8677, Japan; 5 Institute for Integrated Cell-Material Sciences (iCeMS), Kyoto University, Yoshidahonmachi Sakyo-ku, Kyoto, 606-8085, Japan

## Abstract

**Background:**

A reliable extraction technique for resolving multiple spots in light or electron microscopic images is essential in investigations of the spatial distribution and dynamics of specific proteins inside cells and tissues. Currently, automatic spot extraction and characterization in complex microscopic images poses many challenges to conventional image processing methods.

**Results:**

A new method to extract closely located, small target spots from biological images is proposed. This method starts with a simple but practical operation based on the extended morphological top-hat transformation to subtract an uneven background. The core of our novel approach is the following: first, the original image is rotated in an arbitrary direction and each rotated image is opened with a single straight line-segment structuring element. Second, the opened images are unified and then subtracted from the original image. To evaluate these procedures, model images of simulated spots with closely located targets were created and the efficacy of our method was compared to that of conventional morphological filtering methods. The results showed the better performance of our method. The spots of real microscope images can be quantified to confirm that the method is applicable in a given practice.

**Conclusions:**

Our method achieved effective spot extraction under various image conditions, including aggregated target spots, poor signal-to-noise ratio, and large variations in the background intensity. Furthermore, it has no restrictions with respect to the shape of the extracted spots. The features of our method allow its broad application in biological and biomedical image information analysis.

## Background

Biological imaging such as confocal fluorescence microscopy and electron microscopy require the use of protein-labeling techniques to localize individual proteins within cells. Biological markers such as green fluorescence protein [1] and a variety of fluorescent dyes [2,3] for fluorescence microscopy, and colloidal gold [4,5] for electron microscopy are widely used. Molecules labeled with biological markers are generally observed as small specific spots against a background of high brightness. Quantitative comprehension of the localization and statistical distribution of the spots are essential for deciphering biological information. In general, cellular microscopic images have a low signal-to-noise ratio (SNR) and the differences in intensity between signal spot and background are not always clear. Moreover, the texture of those backgrounds is complicated. For these reasons, microscopy images are often difficult to manage computationally. Currently, there are several automatic processing and recognition systems for biological images and they have been applied in the quantitative analysis of biological objects ranging from molecules to cells to whole organisms [6-10].

The purpose of this study was to extract and characterize biological spots of intricate morphology and low contrast in an automatic manner. Current standard techniques for spot extraction consist of edge enhancement for image morphology, including discrete convolution by a high-pass mask and the use of first- or second-order differential operators, based on the magnitude of the spatial differences of the spots [11]. One major problem with this approach, however, results from the blurring and degradation of the image contrast during image acquisition. For some spots with weak contrast, edge extraction is not sufficient. In real-world applications, most biological images contain object boundaries, artifacts, and noise. Therefore, edge enhancement filters may cause difficulties in distinguishing the exact edge of the object's structure from artifacts such as trivial geometric features. Additionally, these techniques can amplify background noise in the image while enhancing the object edge [12,13].

In other methods based on conventional frequency-selective filters [14-18], the precise localization of low-contrast spots may not be possible. High-density areas resulting from the integration of many spots may not allow the isolation of individual spots through frequency-selective filters. In addition, the parameter settings are often so complex as to require their modification whenever the target spot images are changed [19,20]. Furthermore, these methods cannot deal with the varied morphology of the spots.

Spot extraction methods based on conventional mathematical morphology [21] effectively capture the spots' location and their shape information [22-26]. These methods employ a morphological algorithm for background subtraction known as the top-hat transformation [27] or rolling-ball transformation [28]. It is well recognized that the principle of these methods is very effective for extracting a target object from a wide variety of image types [29-34].

Morphological operations use small synthetic images called structuring elements (SEs), which are a fundamental tool in mathematical morphology. The SE used as a probe moves along each pixel of the image. To apply morphological filtering for spot extraction from various types of biological images, the procedure to determine the shape and size of the SE is very important. A commonly used SE shape is the square or disk. In the rolling-ball transformation, a ball-shaped SE (such as a disk SE with weights arranged in order to describe a hemisphere in gray scales) is used. In the above-described methods for spot extraction, these SEs were also used. However, most small contiguous spots cannot be individually distinguished, such that several spots are extracted as one connected region because the size (width) of the SEs is wider than the minimum distance between the peaks of adjacent spots. A suitable SE shape for spot extraction includes a straight-line segment (a fuller description of which is given in the Methods and Results sections); however, since processing by common morphological operations with a single line-segment SE is not isotropic, it cannot consider the geometrical details of an intricate image. Thus, for spot extraction, conventional morphological processing is not effective.

Advanced morphological processing with multiple SEs has been reported [35,36]. In this approach, multiple sets of line-segment SEs generated by rotation of the single line-segment SE in different directions are applied. However, in the discrete space of the images, it is difficult to generate a straight line-segment as the SE that can be rotated in an arbitrary direction. This restriction in the rotational direction of the SE prevents adequate spot detection in complicated biomedical images.

In this study, we solved these problems by introducing a simple and practical approach, an extended mathematical morphology, into the automatic detection of spots in biological images. This technique is based on top-hat transformation with a single SE as the straight line-segment. In our algorithm, an original image can be rotated in arbitrary directions with respect to the single SE. This novel method, which we named rotational morphological processing (RMP), can homogeneously treat with geometrical features in an image under various orientations. Top-hat transformation based on RMP has been applied to spot extraction, in the absence of any hypotheses to fit the spots by 2-D Gaussian shape or minimal intensity. Finally, by isotropic processing with the line-segment SE, contiguous spots can be segmented into individual parts. Our novel method was developed in order to automatically extract spots, such as biological markers consisting of antibodies conjugated with fluorescent molecules, from a biological image of intricate morphology and low contrast.

Smal et al. evaluated the spot detection methods most frequently used in fluorescence microscopy [37], including wavelet-based multiscale detecting [16,18], morphological based methods [23,24,38], and the machine learning method [39]. In this study, we compared the performance of our proposed method with other morphological based methods, such as conventional top-hat transformation and *h*-dome transformation, by using synthetic-noise images.

This report is organized as follows. A brief introduction describing the basics of conventional mathematical morphology is followed, in the Methods section, by a detailed presentation of our spot extraction technique. In the Results section, the application of the detection method to synthetic images as well as to real image data from electron and fluorescence micrographs is discussed. In the final section, the effectiveness of our novel method is summarized and evaluated.

### Conventional mathematical morphology

Mathematical morphology is based on set-theory concepts of the shape of an objective image [21]. An image can be represented by a set of pixels. Morphological operations always deal with a set of two images: an objective image and a SE. Each SE has shape and size characteristics as parameters of the operation. Let *f *denote a gray-scale image function from **Z**^2 ^into [0, *I*-1], where *I *is a positive integer. Let *B *denote a binary SE. The fundamental operators of mathematical morphology are dilation and erosion.

dilation:(1)

erosion:(2)

where *D*_*f *_and *D*_*b *_are the domains of the functions *f *and *B*, respectively. The opening and closing operations are delivered from dilation and erosion.

opening:(3)

closing:(4)

The top-hat transformation is one of the commonly used morphological operations for extracting local bright objects from a low contrast image in gray-scale [27]. It is obtained by subtracting from the original image *f *the opening image *γ*_*B *_using the SE *B*.

Top-hat:(5)

It yields an image in which all the residual features (peaks and ridges) are subtracted by the opening operation. Adding these residual features to the original images has the effect of accentuating objective structures with high intensity [27]. If the difference in intensity between the target objects and the background of the image is markedly small, it is difficult to detect these differences with the human eye. However, these low-contrast objects can be extracted and enhanced by the top-hat transformation.

Another method to extract the local bright object in biological images, based on mathematical morphology, is the *h*-dome transformation [38].

*h*-dome:(6)

where D_*h*_(*f*) is the *h*-dome image of a gray-scale image *f*, (*f*-*h*) represents the result of subtracting a constant value *h *from the gray-scale image, and ρ_*f*_(*f*-*h*) the morphological reconstruction of the gray-scale image from *f*-*h. *The gray-level reconstruction is obtained by iterative geodesic dilation of *f*-*h *under *f *until stability is reached [40].

## Method

### Spot extraction filter: top-hat transformation by RMP

The essential elements required for the spot extraction filter are processing of the biomedical image isotropically and isolation of the adjacent spots from the image background. In order to fulfill these requirements, our method proposes that the objective image can be rotated in arbitrary directions with respect to a single straight line-segment SE whose width has only 1 pixel. The width of this SE ensures determination of the minimum distance between two different spots for isolation. By using this SE, spots separated by distances of only 1 pixel can be distinguished individually. The length of the SE should be adjusted so that it is longer than the size of the target spot. A spot that is smaller than the length of this SE is extracted by the top-hat transformation.

This top-hat transformation by RMP with the straight line-segment SE consists of the following steps:

#### Algorithm 1 (Top-hat transformation by RMP with line-segment SE)

**Figure 1:**
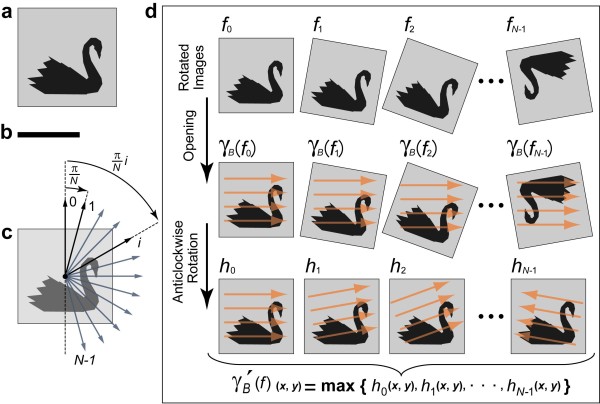
**Schematic procedure of rotational mathematical morphology**. (a) Original image. (b) Straight line-segment SE *B*. (c) Setting of the rotation angles. (d) Process of RMP opening. Original image is rotated and each rotated image is processed by opening with the straight line-segment SE *B*. Orange arrows in each rotated image denote the scan lines by this SE. This rotational processing permits the isotropic processing with a single straight line-segment SE. All processed images (*h*_*i*_) are unified as a result of the RMP opening.

1. *Original image rotation*. The original image *f *(Figure 1a) is rotated in a clockwise direction with respect to the center of the image frame. Assume that dividing a half of the circle (π [rad]) into *N *equiangles gives us each direction at an angle of π/*N *[rad] (Figure 1c), which is an increment angle. Namely, *f*_*i *_(Figure 1d, top row) denotes the rotated image of *f *with the angle of π *i*/*N *[rad], where *i *= 0, 1,..., *N*-1.

2. *Opening*. All rotated images are subjected to an opening operation with the straight line-segment SE *B *(Figure 1b). The opening operations of the rotated image *f*_*i *_are represented as *γ*_*B*_(*f*_*i*_) (Figure 1d, middle row).

3. *Opened image rotation*. The opened images (*γ*_*B*_(*f*_*i*_)) are rotated π·*i*/*N *[rad] in an anticlockwise direction. The rotation at *i *times of the opened image is denoted by *h_i _*(Figure 1d, bottom row).

4. *Union of the rotated and opened images (opening by RMP)*. The processed images (*h*_*i*_) are unified. In union processing, the maximum intensity value, which corresponds to the same pixel coordinate among all opened images, is taken to generate the whole image.

5. *Top-hat transformation by RMP*. The unified opened image (*γ*'_*B*_(*f*)) is subtracted from the original image (*f*).

Figures 1a and 1b show the original image *f *and the straight line-segment SE *B*, respectively. Figure 1c illustrates a setting of the rotation angles. The process in the RMP opening with the straight line-segment SE is represented in Figure 1d. RMP opening is defined as follows:(7)

The image *γ*'_*B*_(*f*)_(*x*, *y*) _is defined as the maximum value of *h*_0 (*x*, *y*)_, *h*_1 (*x*, *y*)_,..., *h*_*N*-1(*x*, *y*)_. The top-hat transformation by RMP is also given by the following equation:(8)

This operation is used as the spot extraction filter in our proposed method.

In this study, we utilized the top-hat transform by RMP for spot extraction from biological images obtained with electron and fluorescence microscopy. The entire practical process consists of the following steps:

#### Algorithm 2 (Spot extraction for practical biological images)

1. *Noise reduction*: Noise, which is less than the resolution limit of the micrograph or target spot, is removed via opening by RMP (equation (7)) with the straight line-segment SE. The length of the SE is set to be smaller than the diameter of the target spots.

2. *Spot extraction*: The spots are extracted by the top-hat transformation by RMP (equation (8)) with the straight line-segment SE. The length of SE is set to be larger than the diameter of the target spots.

3. *Binarization*: The extracted spots are binarized by equation (9) for recognition and measurement of the spots computationally.

*Binarization *is performed according to the thresholding approach:(9)

Namely, the pixels of residual regions by the top-hat transformation by RMP are assigned an intensity of 255 in an 8-bit gray-scale value.

In several cases, the subtraction process and the binarization process leave the small isolated pixels on the image such that they represent residual background noise. The conventional opening operation (equation (3)) can also be applied to remove the remaining noise. This post-processing should be adapted to the particular application.

## Results

### Isolation of overlapped spots

**Figure 2:**
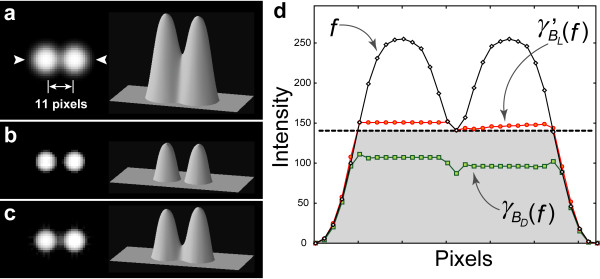
**Isolation of overlapped spots**. (a) Original image of two overlapped spots. Left: original gray-scale image, right: the 3-D map. (b) The result of the top-hat transformation by RMP with the straight line-segment SE *B*_*L *_(11 × 1 pixels). (c) The result of the conventional top-hat transformation with the flat disk SE *B*_*D *_(diameter: 11 pixels). The actual length of 11 pixels is shown in (a). The gray-scale images in (b) and (c) were enhanced by linear contrast stretching. (d) The intensity profiles of the original spots (black line), the result of RMP opening with the straight line-segment SE *B*_*L *_(red line) and of conventional opening with the flat disk SE *B*_*D *_(green line). The position of the profile line is marked by an arrowhead in (a). Residues after subtraction of *f*-*γ'*_*BL*_(*f*) and of *f*-*γ*_*BD *_(*f*) correspond to (b) and (c), respectively. The horizontal black dotted line in (d) denotes the baseline, which is the limit to distinguish between these two spots. The gray region below the baseline is the overlapped region of these spots. To isolate these two spots, the top-surface of the opened image should surpass the baseline.

The performance of our proposed method in the isolation of overlapped spots was compared with that of conventional top-hat transformation. Figure 2a shows the model image of adjacent spots (*f*). On the left of Figure 2a is the original gray-scale image *f *and on the right is the 3-D topographic map of *f*. The vertical height of the map represents the intensity. In top-hat transformation, the original image *f *is first opened and then this opened surface is subtracted from the original surface. In this example, the straight line-segment SE *B*_*L *_(11 × 1 pixels) was used in our method, and the flat disk SE *B*_*D *_(the diameter was 11 pixels) was used in the conventional top-hat transformation. The difference between *f *and its opening by RMP with *B*_*L *_(i.e., top-hat transformation by RMP) is shown in Figure 2b. The result of conventional top-hat transformation with *B*_*D *_of *f *is shown in Figure 2c. In Figure 2d, the black line is the profile of the spots image *f *(the position of the profile line is marked by the arrowhead in Figure 2a), which denotes the surface of the spots. The red line is the result of the opening by RMP with *B*_*L *_of *f *(where the value of the rotational direction *N *is 36). The green line is the result of the conventional opening with *B*_*D*_. Figure 2b shows that the two overlapped spots were segmented clearly by our method.

The opening operation can be geometrically processed by pressing the SE up against the surface of the original image *f *and sliding it underneath the entire surface. The surface of the opened image is constructed from the highest point in the region reached by the SE. Since the line-segment SE *B*_*L *_has a width of 1 pixel, in the process of RMP opening the SE fits the narrow intervening space between the spots. Thus, line-segment SE reaches the baseline of the individual spots (i.e., the upper level of the overlapped region). In contrast, the disk SE *B*_*D *_has a width of 11 pixels as diameter. Since it is larger than the intervening space of the spots, the SE cannot fit the space. Thus, the disk SE cannot reach the level at which the two spots are distinguished. Accordingly, the spots were not isolated by the conventional top-hat transformation.

### Verification of optimal number of rotational direction

The optimal number of the image rotational directions (*N*) was determined. For this experiment, we used a model image containing several artifacts. The image was reconstructed by RMP opening with the straight line-segment SE set at various values for the rotational direction. Peak signal-to-noise ratio (PSNR) was used to provide a quantitative evaluation of this performance. Assuming pixel values in the range of [0: 255], the PSNR is calculated using the following formula:(10)

where *F*(*i*, *j*) denotes the original image, *F*'(*i*, *j*) the filtered image by RMP opening, and *m*_*1 *_× *m*_*2 *_the total number of pixels.

**Figure 3:**
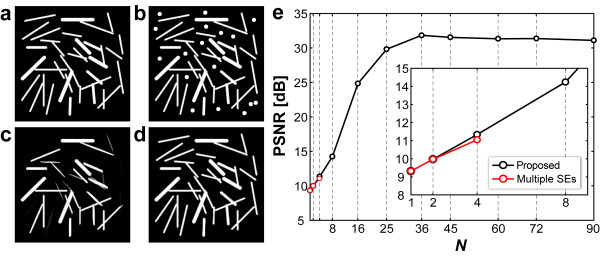
**Verification of the optimal number of rotational directions**. (a) Original image (256 × 256 pixels). (b) Artifact-contaminated image. Some round objects (diameter: 9 pixels) are denoted as artifacts. The image was restored by RMP opening with the straight line-segment SE (31 × 1 pixels) (c) Restored image when *N *(rotational direction number) is 8. (d) Restored image when *N *is 36. (e) PSNR [dB] results of the restored image of (b) by different *N*. The red and black lines show the result of the conventional multiple SEs and our proposed methods, respectively.

Figures 3a and 3b show the original 8-bit gray-scale image (256 × 256 pixels) and the model image overlapped by artifacts, respectively. Some of the round objects (diameter: 9 pixels) are regarded as artifacts to be removed. The RMP opening with the straight line-segment SE (31 × 1 pixels) of *N *= 8 allowed removal of the artifacts but also of some of the elongated target objects (Figure 3c). At *N *= 36, artifacts were removed while the other objects were well preserved (Figure 3d). The graph in Figure 3e shows the variation of PSNR with *N*, which ranged from 0 to 90 directions. The range of small values of *N *(from 0 to 8) is enlarged in the insertion. The black line denotes the result of this operation. The highest value of PSNR was obtained at *N *= 36 and it subsequently remained at almost the same level. In terms of the performance of reconstruction and the computational cost of processing, it is reasonable to assume that the optimal number of image rotational directions is 36.

In addition to this experiment, performance was also tested using the multiple SEs method, shown as a red line in Figure 3e. The straight line-segment SE was used for these tests. In the case of *N *= 1, the single SE (orientation *θ *= 0 [rad], horizontal direction) was applied; at *N *= 2, two SEs (*θ *= 0 and π/2 [rad]) were used, and at *N *= 4, four SEs (*θ *= 0, π/2, π/4 and 3π/4 [rad]) were used. These results showed that reconstruction by the multiple SEs was insufficient.

### Noise reduction

**Figure 4:**
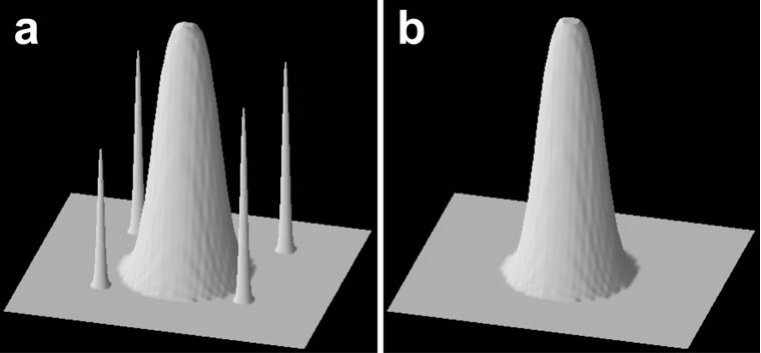
**Noise reduction through RMP opening**. (a) Spot model image with background noises. (b) Noises removed from the image by RMP opening with the straight line-segment SE.

The noise reduction technique in our method (in algorithm 2), i.e., RMP opening with line-segment SE, was used. Figure 4a shows the spot model with background noises. These noises were removed by RMP opening (Figure 4b). The length of the line-segment SE was determined from the spatial resolution of the microscopic image, with objects having a shape size smaller than the resolution limit regarded as noise. If the spot size is known in advance, the SE length should be set to remove objects smaller than the spot size.

### Comparison with conventional morphological methods by using synthetic-noise images

**Figure 5:**
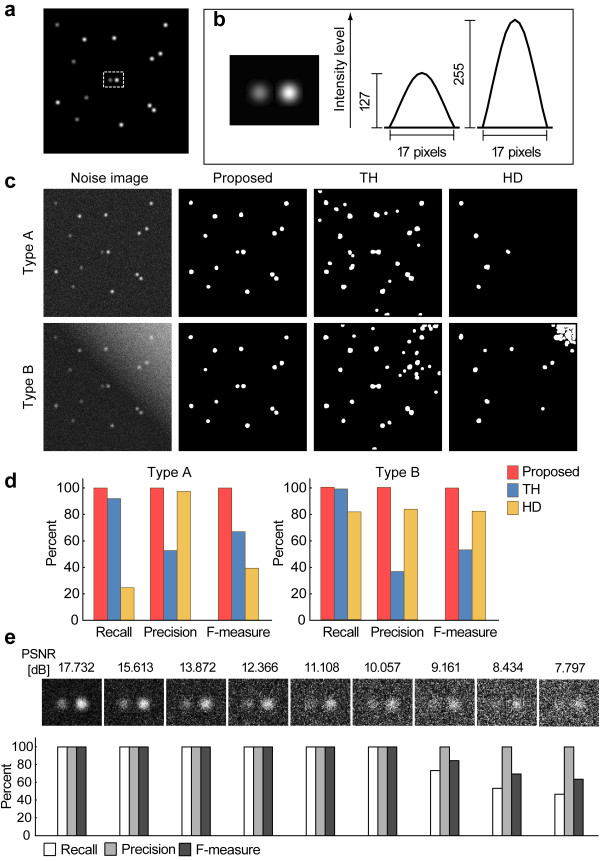
**Experimental results using synthetic images**. (a) Example of source image with an 8-bit gray-scale. (b) 2-D Gaussian distributions as spot models. Two spot models with different intensity levels were used in this experiment. The actual image of the spot models (cropped from the white dotted rectangular region of (a)) are shown on the left, and the intensity profiles on the right. (c) Example results of spot detection using synthetic noise images with a uniform (type-A) and a gradient (type-B) background. The performance of the proposed method was compared with that of conventional top-hat (TH) and *h*-dome (HD) transformation. (d) The recall, precision, and F-measure rates of this experiment. (e) The performance of our method when synthetic images with various noise levels are used.

The ability of the proposed method was compared with that of conventional morphological methods, such as the conventional top-hat and *h*-dome transformations. Synthetic-noise images were generated to evaluate the quantitative performance of spot detection. Ten source images of size 512 × 512 pixels were generated, with one such example shown in Figure 5a. Each image contained 15 spots, which were modeled as a 2-D Gaussian distribution in random locations. This distribution is a good approximation of the theoretical shape of the spot [41]. Two types of spot models with different intensity levels (255 or 127 in an 8-bit gray-scale) were generated (Figure 5b). First, a 2-D Gaussian distribution with kernel width = 17 (pixels) and standard deviation σ = 2 (pixels) was generated. This distribution was then expanded to the normalized gray-scale range of 0-255. For the second spot model, the distribution was expanded to the range of 0-127.

The Poisson-distributed noise was added to each source image with a uniform (type-A) and a gradient (type-B) background (Figure 5c). This is one of the main sources of noise in fluorescence microscopy imaging [42].

The PSNR between each source image and the corresponding noise-added image was calculated. The average value of the PSNR of the type-A image set was 13.848 ± 0.242 (mean ± SD) dB, while for the type-B image set it was 11.245 ± 0.169 dB.

Since the spots in the synthetic image are prospectively known, the recall, precision, and F-measure rates can be calculated. Let TP denote the number of spots detected correctly, FN the number of spots missing detection, and FP the number of spots false alarmed. If adjacent spots are not separated, those spots are regarded as FN. The recall and precision rates are defined by the equations TP/(TP+FN) and TP/(TP+FP), respectively. The F-measure rate is the harmonic mean recall and precision rate and takes into account both measures:(11)

In each image type, the synthetic images had a total of 150 spots; thus, TP + FN = 150. The results detected by our proposed method were compared with those of the conventional top-hat and *h*-dome transformations.

In this experiment, noise-added images were first smoothed with the Gaussian filter (3 × 3 kernel).

Processing by our proposed method followed algorithm 2. Since the width of the spot domain was 17 pixels (Figure 5b), any structure smaller than this width was regarded as noise or artifact. In step 1, the straight line-segment SE of 13 × 1 pixels was used, and in step 2, the straight line-segment SE of 21 × 1 pixels. In both steps, the rotational direction number (*N*) of RMP processing was 36. The subtracted image was binarized by the method in step 3.

For the method based on the conventional top-hat transformation, the processing in step 2 differs from that of the proposed method. Top-hat transformation was applied with disk SE (diameter: 21 pixels) and the subtracted image was binarized by the method in step 3.

For the method based on *h*-dome transformation, this transformation was applied to the smoothed image obtained in step1 of algorithm 2. We set the value of parameter *h *to 50, and the subtracted image (*h*-dome) was binarized by the method in step 3.

Finally, binary images obtained from these methods were cleaned by the conventional opening with disk SE (diameter: 13 pixels) as post-processing.

The results of the spot extraction are seen in Figure 5c. The type-A and type-B images are shown in the top and bottom row, respectively. All spots were correctly detected by our proposed method but not by the other methods. Figure 5d shows the actual values of recall, precision, and F-measure for each type image set. The performance in terms of the F-measure for the proposed method was consistently 100% in all type images. Thus, the proposed method is more tolerant of Poisson noise images than other methods.

In addition, the performance of our method was tested using synthetic images under various noise levels. For the source image (Figure 5a), nine degrees of Poisson-distributed noise images with uniform background were generated, with the PSNR decreasing from 17.732 to 7.797 dB. The upper part of Figure 5e shows the synthetic images, captured only in the rectangular region in Figure 5a. The proposed method was applied to these synthetic images with the same procedure and SEs as in the previous experiment. The bottom of Figure 5e shows that the scores for the recall, precision, and F-measure rates were consistently 100% in the PSNR range of 17.732 to 10.057 dB. Subsequently, the recall rate decreased with decreasing PSNR although the precision rate remained at 100%. This indicates that FP was zero and ensures the accuracy of our method for spot detection.

### Spot extraction of colloidal gold particles

**Figure 6:**
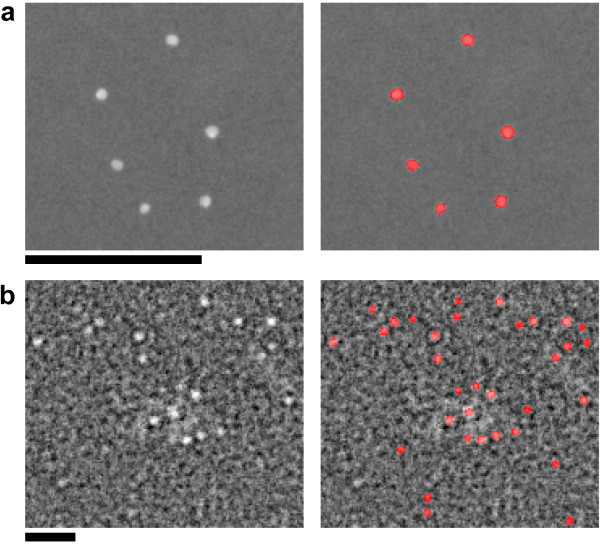
**Spot extraction of colloidal gold particles from electron microscopy images**. (a) Original micrograph of 10-nm-diameter gold particles (left). Spot-extracted image (right). The extracted spots are denoted in red regions. Bar: 200 nm. (b) Original micrograph of 1.8-nm-diameter gold particles (left). Spot-extracted image (right). Scale bar: 10 nm.

The proposed method was applied to electron microscopic images to evaluate its performance in spot extraction. Electron micrographs containing colloidal gold particles were used as test images, with two different sizes of particles (10 nm and 1.8 nm in diameter). Figure 6a (left) shows a part of the original micrograph with 10-nm-diameter gold particles (British BioCell). The spatial resolution was 0.90 nm/pixel. Figure 6b (left) shows a part of the original micrograph with 1.8-nm-diameter gold particles (Nickel (II)-Nitrilotriaceticacid-Nanogold, Nano Probes). The spatial resolution of this image was 0.32 nm/pixel. These images were on an 8-bit gray-scale, the intensity values of the routine image quality were inverted. The purpose of this test was to verify whether the diameter of the extracted gold-particle spot as determined by our method was consistent with the nominal diameter.

Processing of the spot extraction was carried out with algorithm 2. In the case of the micrograph containing 10-nm gold particles, line-segment SE with a size of 5 × 1 pixels (4.5 × 0.9 nm) and 13 × 1 pixels (11.7 × 0.9 nm) was used in noise reduction and spot extraction, respectively. The extracted spots were binarized and overlaid as seen in the red region on the original image. The result is shown on the right side of Figure 6a. For the electron micrograph containing the 1.8-nm gold particles, line-segment SE with a size of 3 × 1 pixels (0.96 × 0.32 nm) and 7 × 1 pixels (2.24 × 0.32 nm) was used for noise reduction and spot extraction, respectively. The result is shown on the right side of Figure 6b. For all extracted particles, the averaged Feret's diameter was calculated. The mean ± SD of the diameter of the extracted spots was 10.16 ± 0.77 nm (164 spots) for the 10-nm gold particles, and 1.81 ± 0.15 nm (813 spots) for the 1.8-nm gold particles. This result shows that the diameter of the extracted spots was consistent with the nominal diameter.

### Spot extraction of fluorescent antibodies

**Figure 7:**
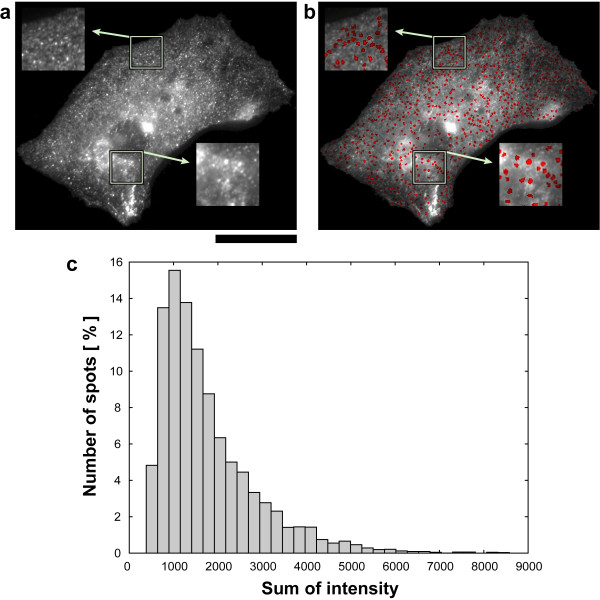
**Quantitative spot extraction of fluorescent antibodies from a fluorescence microscopy image**. (a) Original image. Fluorescence micrograph of caveolin-1 molecules stained with the primary antibody and a secondary fluorescent antibody in normal fibroblasts. Scale bar: 10 μm. (b) The results of spot extraction using the proposed method. These spots were superimposed on the original image as red regions. (c) The histogram of the intensity as estimated from the extracted spots.

Our proposed spot extraction method was applied to fluorescence microscopy images in which caveolin-1 molecules in fibroblasts were stained with secondary fluorescent antibodies (Figure 7a). This image had an 8-bit gray-scale and a spatial resolution of 60 nm/pixel. Line-segment SE with a size of 3 × 1 pixels (180 × 60 nm) and 7 × 1 pixels (420 × 60 nm) was used for noise reduction and spot extraction, respectively.

By visual observation, the diameter of each spot was found to be about 5 pixels (ca. 300 nm). Therefore, a SE length longer than the diameter of the target spots was selected for the spot extraction process. Binarization was carried out using equation (9). The regions of the extracted spots were superimposed on the original image as red-colored regions (Figure 7b). From this micrograph, the 627 spots were extracted. The quantitative estimation of the bright spots corresponding to the caveolae is provided in Figure 7c. The sum of the intensity of the pixel values in each extracted spot region was calculated and the distribution depicted in a histogram. The total number of extracted spots was 6701 from five micrographs (which included approximately 5 cell regions). The median of the histogram was 1319. The largest cluster can be seen centered at the histogram's median value.

**Figure 8:**
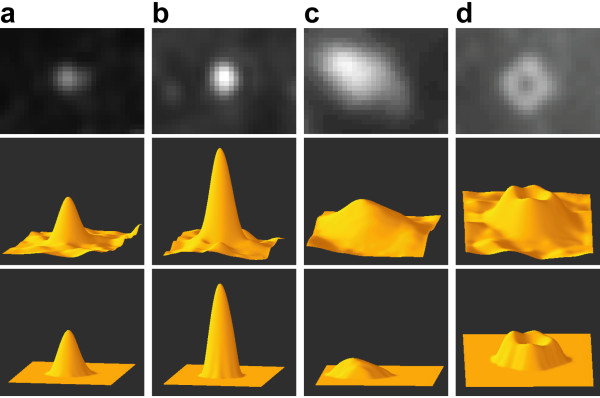
**Precise extraction of various shaped spots**. Examples of the four shapes of fluorescence spots from micrograph are shown in the top row. (a) Gaussian-like shape (low peak height), (b) Gaussian-like shape (high peak height), (c) irregular shape, and (d) volcano shape. The 3-D maps of the original images and of the extracted spots using our proposed method are shown in the middle and bottom rows, respectively.

As shown in Figure 8, the proposed method was applied to the various shapes of the fluorescent spots (top row, original images): (a) Gaussian-like shape (low peak height), (b) Gaussian-like shape (high peak height), (c) irregular shape, and (d) volcano shape. The 3-D maps of the original images are shown in the middle row, which illustrates the relief of the pixel surface. The 3-D maps of the extracted spots, as determined by our method, are shown in the bottom row. All spots were well extracted even though they had intricate shapes.

## Discussion and conclusions

Our novel method to extract the spots in electron and fluorescence microscopic images uses the extended morphological filter through the top-hat transformation by RMP. We have successfully shown that the method is useful for extracting spots in biomedical images in which the conventional method is inadequate. The key concepts of our spot extraction method are the use of a straight line-segment SE and the rotation of the original image. By changing the length of SE, target spots of various sizes can be extracted. The method avoids the technical difficulties of traditional morphological processing and its performance is robust in the processing of biomedical images. The main advantages of our method are that it is computationally simple and easily modified for the extraction of target spots of different sizes and shapes, and that it can handle images in various conditions, e.g., aggregated target spots, poor SNR, and a background with large variations in intensity. The method yields directional information regarding the spatial distribution of spots within the cell as well as the frequency distribution of the size and intensity of the spots.

Our method is based on a line-segment SE with a 1-pixel width as the minimum separation distance and therefore allowed two or more target spots located close to each other to be clearly distinguished (Figure 2). With conventional morphological top-hat transformation using the common SE shape (such as a disk or square), it is difficult to separate such spots. A similar difficulty arises when the "ball" SE is used. Since it has a radius that is larger than the inter-space distance between adjacent spots, it cannot fit within the space. The top surface obtained during opening with the rolling ball cannot reach the baseline allowing for separation of the spots.

To verify the optimality of the number of rotational directions (*N*) shown in Figure 3, we investigated how an artifacts-contaminated image (Figure 3b) could be restored by the RMP opening with increasing *N*. The experimental result (Figure 3e) showed that *N *= 36 was a better trade-off because the value of PSNR was low for *N *< 36 while for *N *> 36 the processing time became longer. In our method, a large computational cost, which is proportional to the size of the input images, is inevitable.

We compared our spot extraction method with the conventional top-hat and *h*-dome transformations. As seen in Figure 5, our method outperformed the others with respect to the three criteria (Figure 5d). For the proposed method, the performance in terms of F-measure rate was maintained at 100% among all background typed images. The precision rate of the conventional top-hat transformation was much lower due to its higher FP value (124 in type-A and 228 in type-B, respectively) in detection of the noise. Furthermore, conventional top-hat transformation could not separate adjacent spots, as it used the disk SE. Meanwhile, the recall rate of the *h*-dome transformation was much lower due to its higher FN value (113 in type-A and 28 in type-B, respectively). Thus, the number of undetected true spots was large.

We further investigated the change in the three measurements as a function of decreasing PSNR from 17.732 to 7.797 dB (Figure 5e). The F-measure rate was maintained at 100% until PSNR decreased to about 10 dB. Subsequently, when PSNR decreased further, the F-measure rate decreased as well due to a decreasing recall rate (thus, increasing the FN value); however, the precision rate was constantly 100%. These results showed that our method is accurate in spot detection.

In the measurement of gold particles in the electron micrograph (Figure 6), the value of the averaged Feret's diameter of the extracted spots and the value of the nominal diameter of the gold particles were in close agreement. Thus, our method effectively extracted spots of the specified size with high accuracy.

Figure 7 shows the location of the small spots in the cell and the estimation of the spots intensities. Previously, Orlichenko reported that stimulation of epithelial cells with epithelial growth factor (EGF) resulted in a profound increase in the number of caveolar structures at the plasma membrane [43]. Our method was able to carry out precise quantitative measurements of the spatial and intensity distributions of the membrane domain with respect to caveolae.

Furthermore, our method allows effective extraction of various shaped spots. Since it is based on the top-hat transformation, the spots are extracted independently in terms of the shape of the surface relief, which is based on variations in the intensity value within a spot region (Figure 8). In the conventional spot detection methods that rely on matched filtering, a 2-D Gaussian distribution is commonly used for the matched filter, assuming that a point-spread function of a signal spot has a 2-D Gaussian distribution. However, because most spots have an irregular topology, as in the example in Figure 8, accurate spot extraction is difficult using the matched filtering method.

Signal spots extracted by our method can be transformed into a 2-D Gaussian distribution as a normalization of spot shape. This allows the application of our method to the conventional automatic tracking system of individual fluorescent particles [44].

The RMP-based method enables a shape and intensity analysis for various types of biomedical images (2-D gel electrophoresis image, DNA microarray image, electron micrograph, X-ray mammographic image, etc.). It can be applied not only to spot extraction but also to a wide variety of important image processing techniques, such as segmentation, smoothing, and pattern extraction [45]. Overall, it provides a wide-ranging analytical approach to biological and biomedical informatics.

## Authors' contributions

YK conceived the study, developed the algorithms, carried out the testing and fine-tuning of the algorithms using the programming language C/C + +, and wrote the first draft of the paper. NB provided useful comments on methodology and helped to revise the manuscript. NM carried out all of the LM, TEM, and molecular biology experiments, supervised the work, and edited and revised the manuscript. All authors read and approved the final manuscript.
